# Mitochondria ubiquitin ligase, MARCH5 resolves hepatitis B virus X protein aggregates in the liver pathogenesis

**DOI:** 10.1038/s41419-019-2175-z

**Published:** 2019-12-09

**Authors:** Young-Suk Yoo, Yeon-Ji Park, Ho-Soo Lee, Nguyen Thi Kim Oanh, Mi-Young Cho, June Heo, Eun-Seo Lee, Hyeseon Cho, Yong-Yea Park, Hyeseong Cho

**Affiliations:** 1 Department of Biochemistry and Molecular Biology, Ajou University School of Medicine, Suwon, 16499 South Korea; 2 Department of Biomedical Sciences, Graduate School of Ajou University, Suwon, 16499 South Korea; 3 Laboratory of Immunogenetics, National Institute of Allergy and Infectious Diseases, NIH, Bethesda, MD USA; 4 MOGAM Institute for Biomedical Research, Gyeonggi-do, 16924 South Korea

**Keywords:** Oncogenes, Mechanisms of disease

## Abstract

Infection of hepatitis B virus (HBV) increase the incidence of chronic liver disease and hepatocellular carcinoma (HCC). The hepatitis B viral x (HBx) protein encoded by the HBV genome contributes to the pathogenesis of HCC and thus, negative regulation of HBx is beneficial for the alleviation of the disease pathogenesis. MARCH5 is a mitochondrial E3 ubiquitin ligase and here, we show that high MARCH5 expression levels are correlated with improved survival in HCC patients. MARCH5 interacts with HBx protein mainly accumulated in mitochondria and targets it for degradation. The N-terminal RING domain of MARCH5 was required for the interaction with HBx, and MARCH5^H43W^ lacking E3 ligase activity failed to reduce HBx protein levels. High expression of HBx results in the formation of protein aggregates in semi-denaturing detergent agarose gels and MARCH5 mediates the elimination of protein aggregates through the proteasome pathway. HBx-induced ROS production, mitophagy, and cyclooxygenase-2 gene expression were suppressed in the presence of high MARCH5 expression. These results suggest MARCH5 as a target for alleviating HBV-mediated liver disease.

## Introduction

The ubiquitin–proteasome pathway is an important system for the processing of abnormally folded or damaged proteins, and failure of protein quality control systems results in the accumulation of cytotoxic protein aggregates. Genetic and environmental factors such as mutations, viral infection, and oxidative stress contribute to the pathogenesis of neurodegenerative diseases and chronic liver diseases^1^. The inability of liver cells to eliminate protein aggregates plays a role in chronic liver diseases such as steatohepatitis and liver cancer^2^. Mallory–Denk bodies (MDBs) are hepatic inclusions containing keratin aggregates, and MDB formation is considered as a failure of protein quality control^3^.

Chronic infection by hepatitis B virus (HBV) is associated with several hepatic diseases ranging from chronic steatosis to hepatocellular carcinoma (HCC)^4^. The HBV x protein (HBx) is a non-structural protein that plays an important role in hepatocytes, promoting the progression of liver disease in patients infected with HBV^5^. HBx exerts a potent transactivation effect, and acts on a wide range of viral and cellular regulatory DNA elements^6,7^. Activation of nuclear factor-κB (NF-κB) and cAMP responsive element-binding transcription factor was activated by HBx leads to uncontrolled cell proliferation^8,9^. Moreover, activation of sterol regulatory element-binding protein 1 (SREBP1) and peroxisome proliferator-activated receptor gamma (PPAR-γ) by HBx induces lipid accumulation in liver cells, as well as in HBx-transgenic mice, leading to HBV-mediated hepatic steatosis^10^. In addition, HBx upregulation is associated with abnormal mitochondrial aggregation and dysfunction^11^. Mitochondrial HBx decreases the mitochondrial membrane potential and increases cellular reactive oxygen species (ROS), promoting oxidative stress and liver inflammation^12–14^. Immunocytochemical staining revealed that HBx forms intracellular aggregates in the cytoplasm and frequently accumulates in large granules in HepG2 cells^15^. Imaging experiments also showed that the cellular amounts of HBx determine its subcellular distribution in the nucleus, cytoplasm, and mitochondria^16^. Development of a protein quality control system for the HBx protein may be useful to decrease the rate of disease progression in patients with chronic HBV infection.

MARCH5/MITOL is one of 11 members of the MARCH family of membrane bound E3 ubiquitin ligases. MARCH family proteins localize to the plasma membrane and to membranes of intracellular organelles, such as the endosome, endoplasmic reticulum (ER), and mitochondria^17^. MARCH5 localizes to the outer membrane of mitochondria and plays an important role in the maintenance of mitochondrial homeostasis. MARCH5 regulates mitochondrial dynamics by ubiquitinating the mitochondrial proteins Drp1, Fis1, and Mfn1^18–20^. MARCH5 is involved in protein quality control, and specifically recognizes and binds to mutated superoxide dismutase-1 (SOD-1) and expanded polyglutamine aggregates that accumulate in mitochondria^12,21,22^. In addition, MARCH5 recognizes and targets functional MAVS aggregates, which are important for the innate immune response, for degradation, thereby preventing persistent and harmful immune responses^23^. The mechanism by which MARCH5 preferentially binds oligomerized or aggregated proteins over monomeric substrates remains unknown; however, this particular feature provides potential therapeutic options in diseases related to protein aggregation.

In the present study, we showed that MARCH5 targets HBx protein aggregates and promotes proteasome-mediated HBx degradation. MARCH5 may attenuate hepatic inflammation by suppressing HBx-induced ROS production and cyclooxygenase-2 (COX-2) gene expression. The present findings suggest that MARCH5-mediated HBx degradation is important for preventing severe liver pathogenesis.

## Results

### MARCH5 expression is positively correlated with the survival rate of patients with liver tumors

**Fig. 1:**
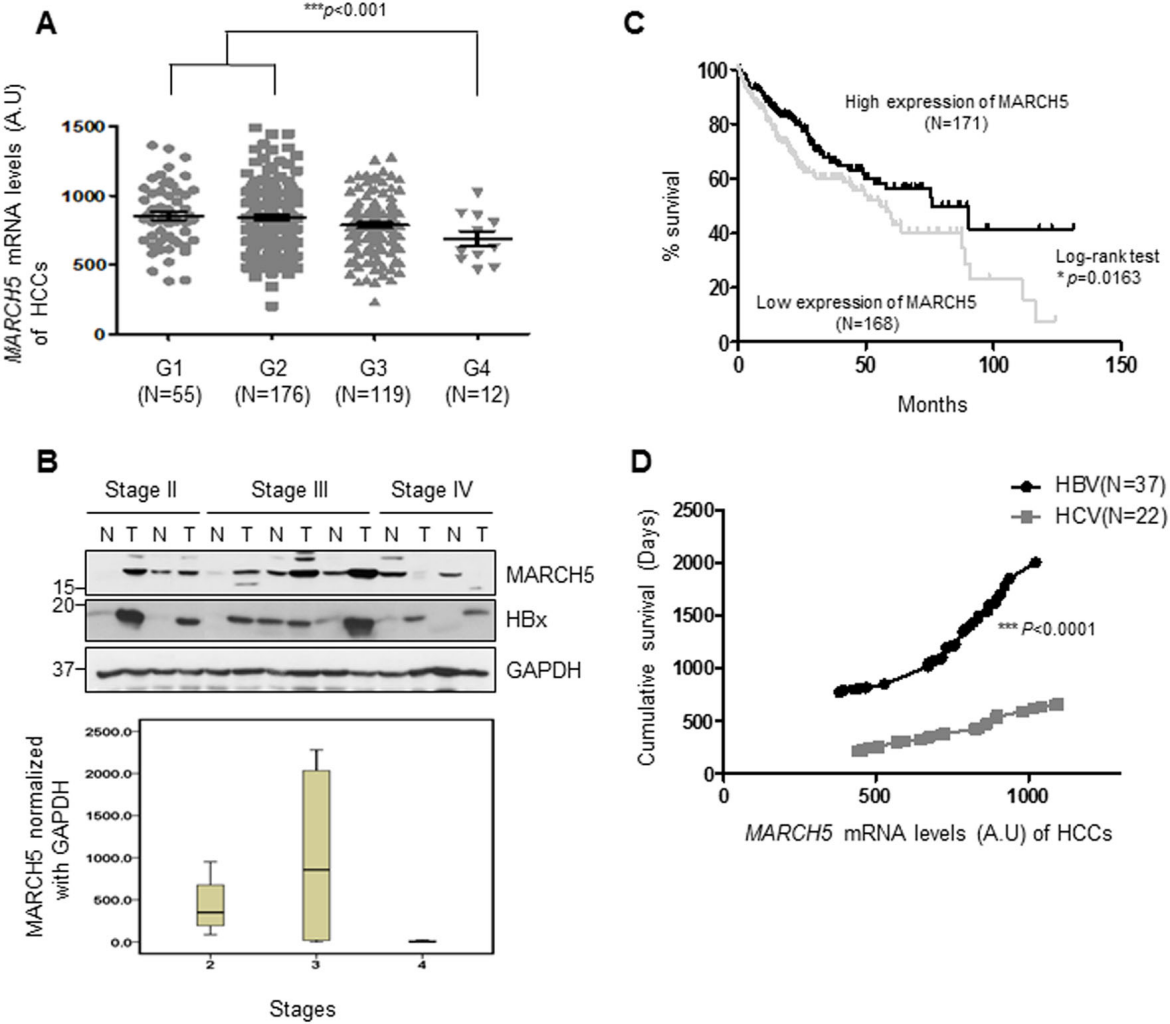
Analysis of MARCH5 mRNA and protein expression in HCC tissues. **a** Analysis of MARCH5 mRNA expression in histologic grades of human hepatocellular carcinomas (HCCs) of TCGA dataset (***p < 0.001; Wilcoxon’s signed-rank test). **b** Western blot analysis of MARCH5 and HBx protein expression in human HCCs (T) and matched surrounding non-tumor liver tissues (N). Densitometric values of MARCH5 normalized with GAPDH (lower panel). **c** The survival rates of HCC patients by MARCH5 mRNA expression levels. Analyzed by a Kaplan–Meier curve (**p = 0.0163, Log-rank (Mantel–Cox) test). **d** The cumulative overall survival days of HCC patients (G2 and G3) with HBV infection or HCV infection. MARCH5 mRNA expression in HCC patients with HBV infection was correlated with cumulative survival (***p < 0.001; Mann–Whitney test).

MARCH5 is a crucial regulator of mitochondrial dynamics and protein quality control^19^. Despite the role of aberrant protein quality control in the pathogenesis of various diseases, the expression and function of MARCH5 in liver cancers remain undetermined. Here, we first analyzed MARCH5 expression levels in HCC tissue specimens from The Cancer Genome Atlas (TCGA) database. Cancer tissue specimens were classified into four Grades (G1–G4) according to the American Joint Committee on Cancer histologic grade classification of HCC tumors. MARCH5 mRNAs were accumulated in HCC tissues. MARCH5 mRNA levels in low Grade tumors (G1-G2, *N* = 231) were not different from those in Grade 3 tumors (*N* = 119). However, we found that MARCH5 mRNA levels in Grade 4 tumors (*N* = 12) were significantly downregulated compared to those in lower Grades tumors (*P* < 0.001; Wilcoxon’s singed-rank test, Fig. 1a). These findings were confirmed by measuring MARCH5 protein levels in HBV-related HCC specimens obtained from Ajou Hospital in Korea. Liver tissue specimens from HCC patients were collected as pairs of tumors and surrounding non-tumor tissues, and tumors were classified into four stages (Stage I–IV) according to the tumor-node-metastasis classification for HCC by the International Union against Cancer. MARCH5 protein expression was higher in Stage II and III tumor specimens than those in surrounding non-tumor tissues (Fig. 1b). In contrast, MARCH5 protein levels were remarkably decreased in Stage IV tissues, consistent to MARCH5 mRNA expression (Fig. 1a), while MARCH5 protein expression in surrounding non-tumor tissues of Stage IV remained unchanged. We also observed that HBx protein expression in HCCs was significantly higher than those in surrounding non-tumor tissues. In Stage IV, an inverse relationship between MARCH5 and HBx was found in both tumors and surrounding non-tumor liver tissues (Fig. 1b). This suggested that MARCH5 function is lost in stage IV malignant liver tumors. TCGA data were used to analyze the role of MARCH5 expression in HCC patient outcomes, which showed that MARCH5 mRNA expression was positively correlated with patient survival (Fig. 1c). Kaplan–Meier analysis indicated that patients with high MARCH5 mRNA expression levels had better survival than those with low MARCH5 expression (*p* = 0.0163; Log-rank (Mantel–Cox) test, Fig. 1c). We next compared the MARCH5 mRNA expression level in HBV-related HCCs and HCV-related HCCs using the TCGA database and found that MARCH5 mRNA expression levels were similar in HBV-related HCCs and HCV-related HCCs (Fig. S1). Importantly, however, we found that MARCH5 mRNA levels are only well correlated with cumulative survival in patients with HBV-related HCCs, suggesting that some factors derived from the HBV infection is affected by MARCH5 (*p* < 0.0001; Mann–Whitney test, Fig. 1d). Taken together, these data suggest that MARCH5 is involved in survival of HCC patients.

### MARCH5 interacts with the HBx viral oncoprotein

**Fig. 2:**
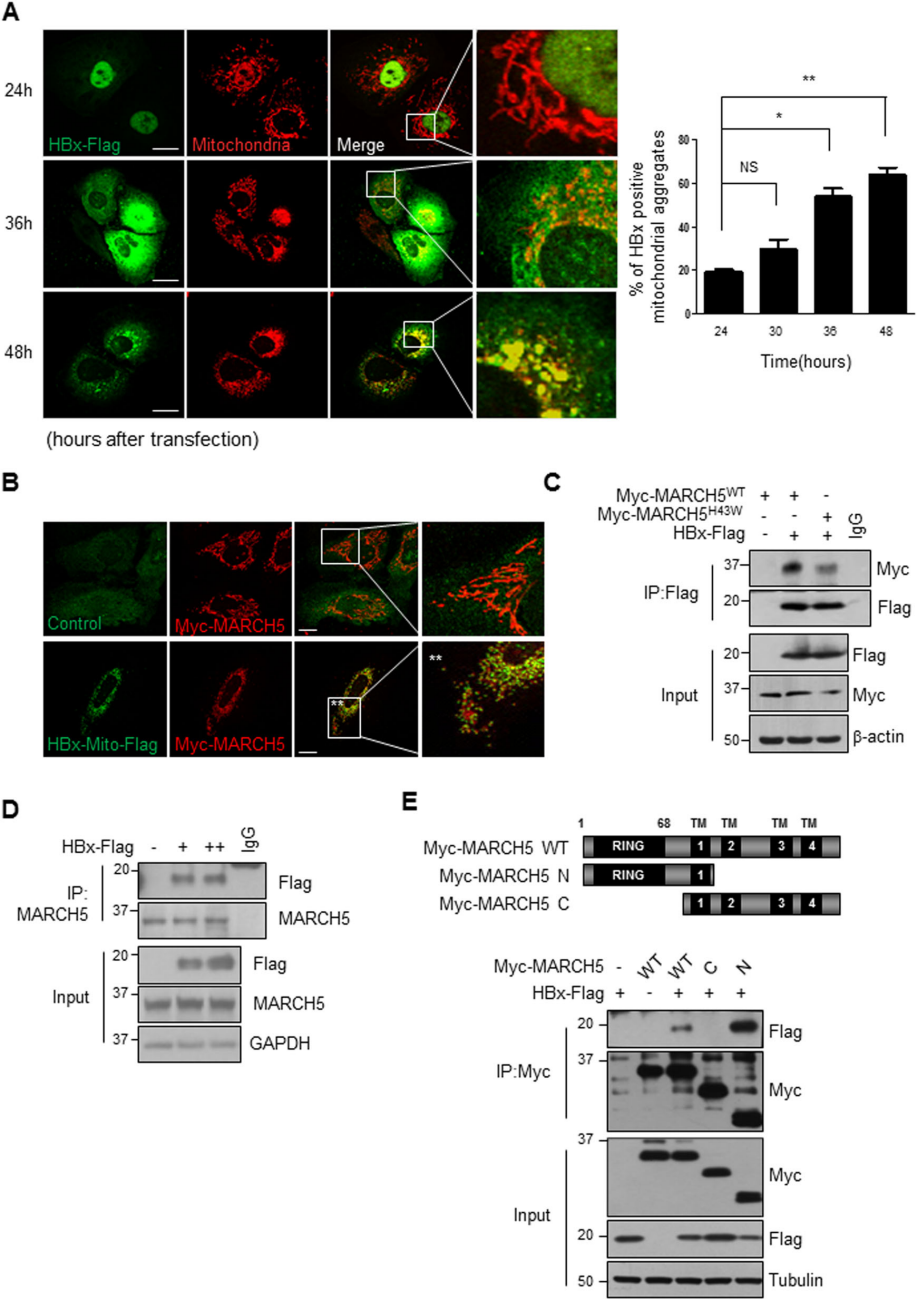
MARCH5 interacts with HBx. **a** After transfection of HBx-Flag for indicated times, subcellular localization of HBx was determined by immunostaining with anti-Flag antibody, followed by FITC-conjugating secondary antibody. Mitochondria was co-stained using MitoTracker -Red. The number of HBx-positive mitochondria aggregated cells were quantified and shown in the right panel (*p < 0.05, **p < 0.01, Two-tailed paired Student’s *t*-test). **b** Huh7 cells were transfected with Myc-MARCH5^WT^ and HBx-Mito-Flag. After 36 h post-transfection, cells were stained by immunostaining with anti-Myc antibody and anti-Flag antibody. **c** After HEK293T cells were transfected with Myc-MARCH5^WT^, Myc-MARCH5^H43W^, and HBx-Flag, cell lysates were immunoprecipitated with anti-Flag antibody and immunoblotted with anti-Flag or anti-Myc antibody. **d** HEK293T cells were transfected with HBx-Flag and lysed cell extracts were immunoprecipitated with anti-MARCH5 antibody. **e** A schematic diagram showing MARCH5 deletion constructs (upper panel). In the lower panel, Huh7 cells were co-transfected with HBx-Flag and Myc-MARCH5^WT^ or MARCH5 deletion constructs. The cell lysates were immunoprecipitated with anti-Myc antibody and immunoblotted with anti-Flag antibody.

Because mitochondrial MARCH5 is critical for the maintenance of mitochondrial and cellular homeostasis, we examined whether the HBx viral oncoprotein is a target of MARCH5 in liver cells. First, the subcellular localization of HBx in Huh7 cells was examined after transfection of HBx-Flag because the subcellular localization and function of the HBx protein are variable. Confocal microscopy showed that, at 24 h after transfection, HBx was detected mostly in the nucleus and partly in the cytoplasm (Figs. 2a and S2a). HBx accumulated in the cytoplasm and mitochondria at 36 h, whereas at 48 h, more than half of the HBx protein was detected as dot-like aggregates in mitochondria (Fig. 2a, lower panel). The mitochondria in Huh7 cells at this time-point were no longer connected, and fragmented mitochondrial morphology was predominant compared with that at 24 h. These findings were consistent with those of previous reports, and indicate the presence of damaged mitochondria with a low mitochondria membrane potential and increased ROS generation^12,21^. Ectopically expressed mitochondria-targeted HBX (HBx-Mito-Flag) containing the targeting sequence for the mitochondrial outer membrane from the TOM protein almost completely colocalized with Myc-MARCH5 and resulted in fragmented mitochondria (Fig. 2b). We showed that HBx-mutant deleting mitochondria target sequences (HBx ΔMTS) do not affect mitochondria morphology (Fig. S2c). However, ectopic expression of Myc-MARCH5 had no significant effect on mitochondrial morphology. Co-immunoprecipitation experiments were performed next to determine whether MARCH5 binds to HBx. The results showed that HBx-Flag bound to wild-type MARCH5 (MARCH5^WT^) as well as to the mutant MARCH5^H43W^, in which the histidine residue in the RING domain is substituted by tryptophan (Fig. 2c). Furthermore, co-immunoprecipitation experiment using anti-MARCH5 antibody verified that endogenous MARCH5 interacts with HBx protein (Fig. 2d). We next mapped the binding domain of MARCH5 using deletion mutants of MARCH5. Co-immunoprecipitation using an anti-Myc antibody showed that a deletion mutant of MARCH5 (MARCH5 N) containing the RING domain and the first transmembrane domain retained strong binding to HBx (Fig. 2e). By contrast, MARCH5 C containing the four transmembrane domains of MARCH5 showed no binding to HBx. Immunofluorescence staining data showed that the MARCH5 C (ΔRING) mutant fully localizes to mitochondria. On the other hand, the MARCH5 N (ΔTM 234) mutant containing one transmembrane domain locates both in the mitochondria and in cytoplasm (Fig. S2b). Taken together, these results indicated that HBx protein aggregates accumulate in mitochondria and cause mitochondrial damage, and the mitochondrial E3 ligase MARCH5 may target HBx for degradation for protein quality control.

### MARCH5 promotes the degradation of HBx protein aggregates

**Fig. 3:**
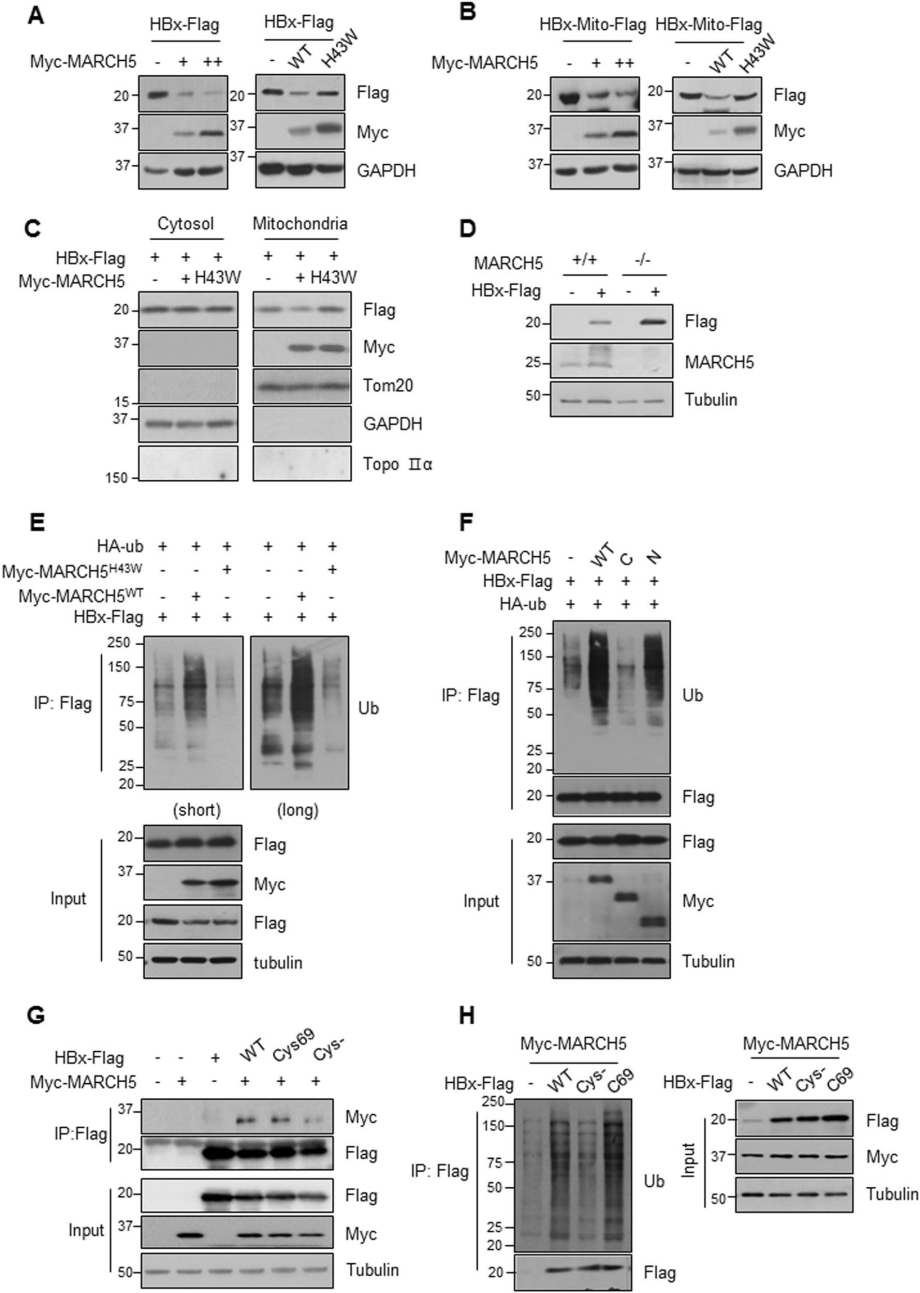
MARCH5 degrades HBx protein. **a**, **c** Huh7 cells were transfected with either HBx-Flag or HBx-Mito-Flag along with Myc-MARCH5^WT^ and expression of HBx protein was analyzed by immunoblotting. **b** The HBx-Flag plasmid was transfected into HEK293T cells or MARCH5 knockout HEK293T cells (*MARCH5*^−/−^). Expression levels of HBx protein in these cells were detected by immunoblotting. **d** After transfection, cells were homogenized and mitochondria fraction and cytosolic fraction were separately obtained. Expression of HBx protein was analyzed by immunoblotting. **e** Cells were transfected with HA-Ub, HBx-Flag, Myc-MARCH5^WT^, and MARCH5^H43W^. The cell lysates were immunoprecipitation with anti-Flag antibody and analyzed by immunoblotted with anti-Ub antibody. **f** Cells were co-transfected with indicated constructs. The HBx-Flag ubiquitination was monitored by immunoprecipitation with anti-Flag antibody and analyzed by immunoblotting with anti-Ub antibody. **g** Cells were transfected with indicated constructs and immunoprecipitation was carried out. **h** Ubiquitination patterns of HBx mutants were analyzed using anti-Ub antibody.

Next, we examined whether the MARCH5–HBx interaction is involved in the quality control of the HBx protein in mitochondria. MARCH5 significantly downregulated the HBx protein in a dose-dependent manner in Huh7 cells co-transfected with HBx-Flag or HBx-Mito-Flag and Myc-MARCH5 expression vectors. Consistently, co-expression of MARCH5 significantly downregulated HBx-Flag and HBx-Mito-Flag. HBx was dependent on its RING domain, because the E3 ligase defective MARCH5^H43W^ mutant did not decrease HBx protein levels (Fig. 3a, b). We compared the degradation of HBx proteins in cytosolic faction as well as in the mitochondria fraction. In the mitochondria fraction, the HBx protein level was reduced by wild type of MARCH5 expression but with MARCH5^H43W^ mutant (Fig. 3c). Furthermore, we addressed whether the depletion of MARCH5 affects the expression level of HBx. We found that HBx level was accumulated in MARCH5 knockout (KO) HEK293T cells (Fig. 3d). An in vivo ubiquitination assay was performed in HEK293T cells transfected with HBx-Flag and Myc-MARCH5 expression vectors along with the HA-ubiquitin (HA-Ub) plasmid. The HBx protein was co-immunoprecipitated using an anti-Flag antibody, and ubiquitination patterns were examined by immunoblotting using an anti-ubiquitin antibody. Ectopic expression of Myc-MARCH5 increased the levels of ubiquitin-conjugated HBx in a dose-dependent manner (Fig. S3a). The in vitro ubiquitination assay showed that MARCH5 acted together with an E1 ubiquitin-activating enzyme and the UbcH5b E2 ubiquitin-conjugating enzyme and effectively transferred ubiquitin to HBx (Fig. S3b). The E3 ligase activity of MARCH5 was required for HBx ubiquitination, because ubiquitin-conjugated HBx was not detected in cells expressing MARCH5^H43W^ (Fig. 3e). MARCH5 N which retained the ability to bind HBx, increased the levels of ubiquitinated HBx (Fig. 3f). These data demonstrated that the MARCH5 E3 ligase polyubiquitinated and decreased the levels of the HBx protein.

The cysteine residues of HBx form inter-disulfide and intra-disulfide bonds, and formation of disulfide bonds and hydrophobic interactions between HBx monomers induce the oligomerization of HBx and the formation of protein aggregates. We previously showed that the complete loss of cysteine residues on HBx (HBx-Cys^−^) results in the formation of only the monomeric form of HBx under non-reducing conditions, whereas HBx-C69, containing a single cysteine at amino acid residue 69, generates not only HBx monomers, but also HBx dimers and oligomeric aggregates^24^. Because MARCH5 preferentially binds protein oligomers or protein aggregates, we investigated whether MARCH5 favors oligomeric HBx binding. Co-immunoprecipitation of Myc-MARCH5^WT^ and HBx Wild type and mutants showed that MARCH5 bound to HBx-C69 as efficiently as to HBx-WT, whereas MARCH5 binding to HBx-Cys^−^ was significantly reduced (Fig. 3g). Consistently, ubiquitin conjugation was significantly lower in HBx-Cys^−^ than in HBx-WT and HBx-C69 in the in vivo ubiquitination assay (Fig. 3h). Taken together, these data suggest that MARCH5 promotes the degradation of HBx protein aggregates.

### MARCH5 resolves HBx aggregates through the proteasome-dependent degradation pathway

**Fig. 4:**
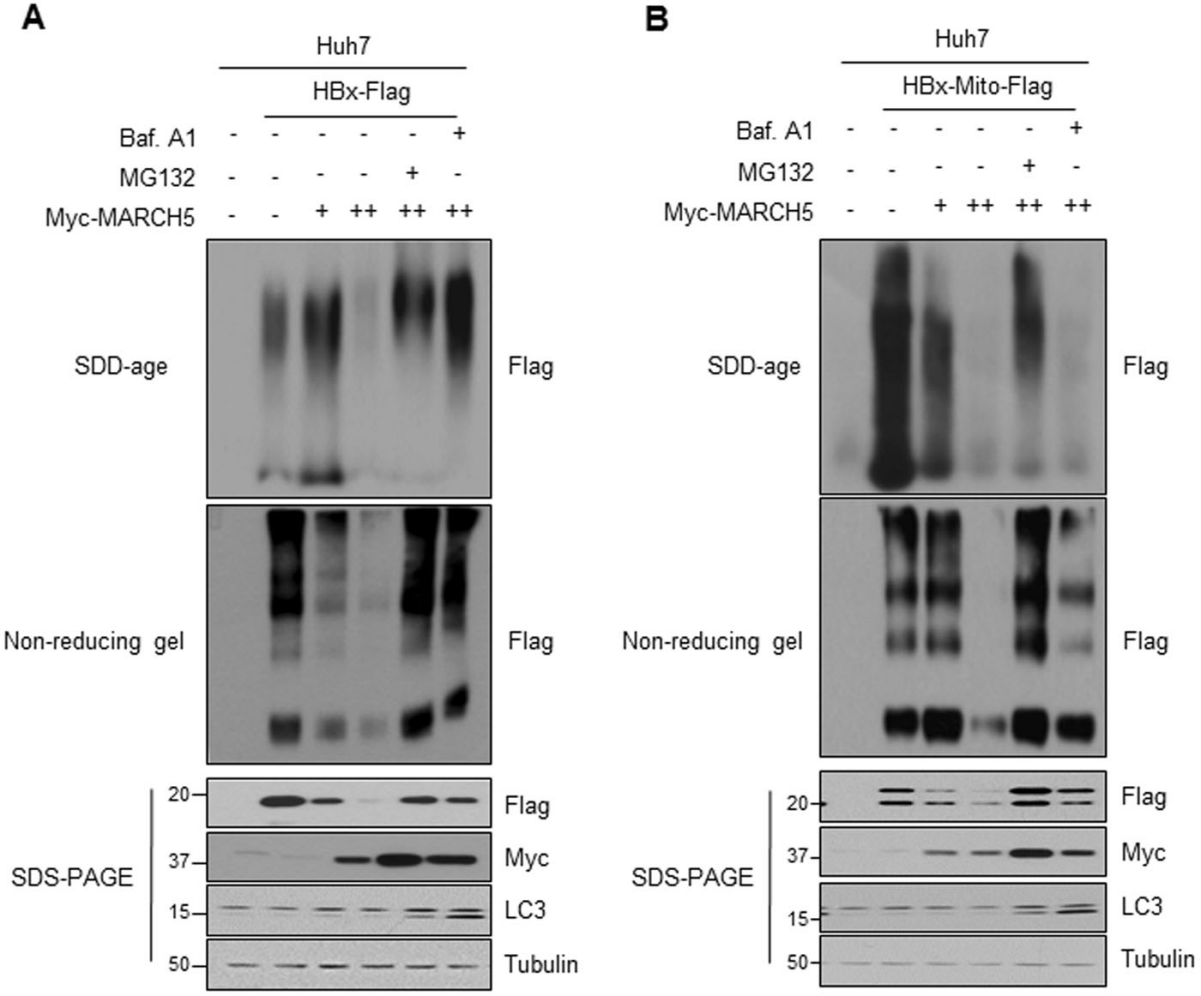
MARCH5 promotes degradation of HBx aggregates in proteasome- and autophagy -dependent manners. **a**, **b** Huh7 cells were transfected with HBx-Flag or HBx-Mito-Flag along with increasing concentrations of Myc-MARCH5 for 36 h. Before harvest, cells were treated with 20 μM of MG132 or 2.5 nM of bafilomycin A1 (Baf A1) for 12 h. To detect HBx aggregates, the cells were directly lysed with sample buffer without β-mercaptoethanol and analyzed on the SDD-AGE (semi-denaturing detergent agarose gel electrophoresis). Alternately, the cell lysates were incubated with 1 mM of DSS as a cross-linker for 30 min and quenched with 10 mM Tris–HCl. Then, the sample buffer without SDS and β-mercaptoethanol was added and analyzed on the SDS–PAGE.

Protein quality control in cells is primarily carried out by the ubiquitin–proteasome system (UPS). The stability of the HBx protein is regulated by several proteins including p53 and the Siah E3 ligase in a proteasome-dependent manner^25,26^. We showed that HBx accumulated in mitochondria triggered extensive mitochondrial fragmentation (Fig. 2b), and cytotoxic substances that accumulate in mitochondria can be eliminated by autophagy^27^. We examined whether the degradation of HBx aggregates by MARCH5 is mediated by the proteasome pathway. Full-length HBx-Flag and Myc-MARCH5 were co-transfected into Huh7 cells, which were treated with the proteasome inhibitor MG132 or autophagy inhibitor, Bafilomycin A1 (Baf A1). We found that MARCH5 expression significantly downregulated HBx, and this reduction was prevented by both proteasome-dependent and autophagy-dependent pathways (Fig. 4a, lower panel). Similarly, the downregulation of the Mito-HBx protein by MARCH5 was suppressed by exposure to MG132, but not Baf A1 (Fig. 4b, lower panel). These results suggested that Mito-HBx was mainly degraded through the ubiquitin–proteasome pathway. We also examined the effect of MARCH5 on HBx and Mito-HBx aggregates. Semi-denaturing detergent agarose gel electrophoresis (SDD-AGE) and non-reducing gel showed that both HBx-Flag and HBx-Mito-Flag formed protein aggregates, and MARCH5 significantly reduced these HBx aggregates (Fig. 4a, b, upper panel). The MARCH5-mediated reduction of HBx aggregates was suppressed in the presence of MG132. These results suggest that HBx forms protein aggregates in cells, and MARCH5 resolves HBx aggregates through the proteasome-dependent degradation pathways.

### MARCH5 attenuates hepatic inflammation induced by the HBx protein

**Fig. 5:**
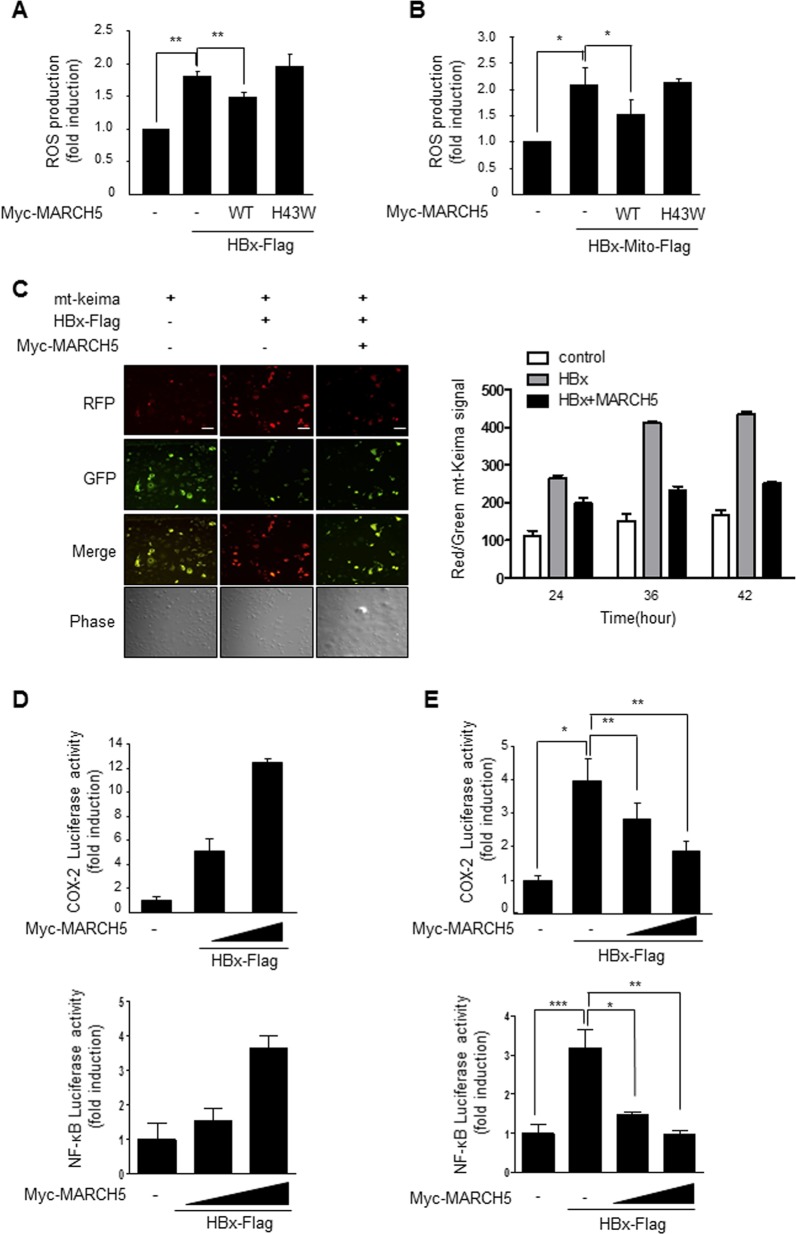
MARCH5 decrease the HBx- induced COX-2 and NF-κB activities. **a**, **b** Huh7 cells were transfected with indicated constructs for 36 h. Intracellular ROS levels were determined by incubation of cells with DCF-DA for 20 min at 37 °C, followed by flow cytometry. **c** Huh7 cells were co-transfected with indicated constructs along with mt-Keima. Fluorescence images were analyzed by confocal microscopy. Scale bar, 100 μm. Graph indicates the Red/green mt-Keima signal. **d**, **e** Huh7 cells were co-transfected with COX-2 luciferase reporter or NF-κB luciferase reporter along with indicated constructs. Luciferase activity was determined. Graphs represent fold-induction of the luciferase activity. Error bars represent the mean ± s.e.m. (*n* = 3).

COX-2 plays an important role in inflammatory responses, and hepatic inflammation contributes to HBV-associated liver carcinogenesis. We previously showed that mitochondrial damage induced by HBx increases ROS levels and COX-2 gene expression, and HBx-mediated COX-2 gene expression requires ROS as well as cytoplasmic calcium signaling^12^. We tested whether MARCH5 expression alleviates HBx-mediated pathogenic signaling. Consistent with previous findings, HBx and Mito-HBx increased ROS levels, and co-expression of MARCH5 suppressed the increase of ROS levels in Huh7 cells (Fig. 5a, b). The MARCH5^H43W^ mutant lacking E3 ligase function had no effect on the HBx-mediated increase of ROS. The fluorescent dye Mito-Keima (mt-Keima) was used to assess mitochondrial damage in HBx-expressing cells. Damaged mitochondria are selectively removed by mitophagy, which can be monitored using the pH-sensitive Keima protein^28^. We introduced the HBx gene into Huh7 cells along with mt-Keima, and imaging of Red fluorescence was performed using the confocal microscopy. At 24 h after transfection, RFP fluorescence appeared in Huh7 cells, and significantly increased at 36 h. Co-expression of MARCH5 decreased the number of cells expressing RFP, suggesting that mitochondrial damage induced by HBx was attenuated by overexpression of MARCH5. (Fig. 5c). This suggested that MARCH5 alleviates HBx-mediated mitochondrial damage. Next, a COX-2 and NF-κB luciferase reporter plasmid was used to investigate the effect of HBx on COX-2 and NF-κB expression. HBx increased COX-2 and NF-κB luciferase activity in a dose-dependent manner (Fig. 5d), and co-expression of MARCH5 significantly suppressed HBx-induced COX-2 and NF-κB luciferase activity (Fig. 5e). Next, we used clonogenic cell proliferation assays to investigate whether the HBx accumulation by the MARCH5 knockdown exerted an effect on colony formation. After determining expression levels of HBx-Flag among different clones by Western blotting. We showed that accumulation of HBx protein in MARCH5 knockdown cells, which is accompanied with increase in colony formation (Fig. S4). Collectively, the data indicated that MARCH5 alleviated HBx-mediated inflammatory signaling by reducing HBx-mediated ROS production and mitochondrial damage.

## Discussion

HBx contributes to the pathogenesis of HCC through its role in transcriptional transactivation and DNA repair, and its effect on epigenetic changes and apoptosis^27,29–33^. Overexpressed HBx forms protein aggregates that predominantly localize to mitochondria, causing mitochondrial damage and increasing ROS levels^12,27^. A cellular quality control mechanism is necessary to attenuate HBx-induced mitochondrial toxicity. In the present study, we demonstrated that MARCH5 regulates HBx protein aggregates. Our findings can be summarized as follows: (i) MARCH5 binds to and targets HBx for degradation by mediating its ubiquitination in mitochondria. Increased levels of MARCH5 therefore attenuate HBx-induced mitochondrial toxicity. (ii) TCGA analysis showed that MARCH5 mRNA expression was strongly correlated with patient survival in HCC. This indicates that MARCH5 expression in hepatocytes is important for the maintenance of cellular homeostasis and for preventing the development of malignant liver tumors.

HBx showed a diverse intracellular distribution pattern and was detected in the nucleus, cytoplasm, and mitochondria. Ectopically expressed HBx was initially localized to the nucleus, translocating to the cytoplasm and finally to mitochondria in a time-dependent manner. We and others reported that high expression levels of HBx result in abnormal mitochondrial morphology and function^11^. HBx-associated mitochondrial damage causes mitochondrial dysfunction by increasing ROS production, voltage-dependent anion channels (VDAC), inducing the activation of NF-κB and STAT3 or translocation of Raf1 to mitochondria^12,14,34–37^. We previously showed that COX-2 induction was correlated with the ability of HBx to increase ROS levels^12^. A mitochondrial localization-defective HBx mutant did not increase intracellular ROS levels or induce COX-2 expression, and exposure to antioxidants or ectopic expression of manganese SOD or catalase abolished HBx-mediated COX-2 induction. In addition to antioxidants, activation of the UPS by MARCH5 may alleviate liver pathogenesis in patients infected with HBV^24^.

The UPS is essential for maintaining cellular homeostasis through its role in protein quality control. MARCH5 is a mitochondrial E3 ubiquitin ligase involved in mitochondrial quality control^19^. MARCH5 localizes to the mitochondrial outer membrane and modulates the levels of target proteins, thereby ensuring that adequate levels of protein are available for cellular needs and function. MARCH5 controls mitochondrial morphology by catalyzing the ubiquitination of Mfn1 under stress conditions and regulates tethering of the ER to mitochondria by ubiquitinating Mfn2^38^. MARCH5 removes abnormal proteins to maintain the cellular homeostasis. MARCH5 promotes the elimination of abnormal mutated or aggregated proteins, poly Q, or mutated SOD^21,22^. Here, we elucidated the mechanism underlying the function of MARCH5 in protein quality control against HBx aggregates. The present study is the first to show that the quality control function of MARCH5 is involved in tumor progression. Somatic mutations of the MARCH5 gene are found in cancer cell lines and tissues. Substitution of tryptophan by histidine 43 in the RING domain of MARCH5 was reported in cancer tissues. MARCH5 has a C3HC4 zinc finger motif in the RING domain, and the RING domain histidine is a conserved residue involved in zinc coordination and important for ligase activity^20^.

## Material and methods

### Cell culture and transfection

The Huh7 (RRID:CVCL_0336), the Chang (RRID:CVCL_0238), which is a HeLa derivative and the HEK293T (RRID:CRL-1573) cells were cultured in Dulbecco’s modified Eagle’s medium (DMEM; Invitrogen) supplement with 10% heat-inactivated fetal bovine serum (FBS) and 1% penicillin–streptomycin (GIBCO BRL) in a 5% CO_2_ incubator at 37 °C. Huh7 is a well-differentiated hepatocyte-derived cellular carcinoma cell line. The Chang (HeLa derivative) and HEK293T were originally obtained from the American Type Culture Collection (ATCC) and the Huh7 cell line was purchased from the Korean Cell line Bank (KSLB, Korea). All these cell lines have been authenticated using the STR profiling by the ATCC. Plasmid DNA transfections were carried out using polyethylenimine (PEI; Polysciences) according to the manufacturer’s instructions. DNA was mixed with PEI in a ratio of 1:2.5 and incubated in OPTI-MEM for 15 min at room temperature. The DNA complex was directly added to cells and incubated for 36–48 h to allow gene expression.

### Generation of MARCH5 KO cells

MARCH5 knockout HEK293T cells were previously generated using transcription activator-like effector nuclease (TALEN) technology. The MARCH5-specific TALEN plasmids were obtained from ToolGen, Inc.^23^

### Plasmids and construction

HBx-Flag and HBx-Mito-Flag plasmids were established^12^ and used in this study. Myc-tagged MARCH5-WT and MARCH5-H43W were established as previously described^18^. The MARCH shRNA plasmid targeting 3′-UTR sequences previously established was used in this study.^39^

### Liver tissue specimens

Paired specimens (tumor and surrounding non-tumor tissues) were obtained from patients with HCC whom underwent hepatectomy at Ajou University Hospital and enrolled to the Ajou Human Bio-Resource Bank. Clinicopathological features of patients were analyzed and were previously described^24^. All patients were chronic carriers of HBV and tumor stages were determined according to a modified UICC staging system. The biospecimens and data used for this study were provided by the Biobank of Ajou University Hospital, a member of Korea Biobank Network.

### Antibodies

The following antibodies were used in immunoblotting analysis. Anti-MARCH5 (1:1000) was from Abcam. Anti-FLAG (M2; 1:2000) was from Sigma. Antibodies for anti-c-Myc (1:1000), anti-Ub (1:1000), anti-tubulin (1:1000), anti-GAPDH (1:1000), TopIIα (1:1000), Tom20 (1:1000) were purchased from Santa Cruz Biotechnology.

### Immunoblot analysis and immunoprecipitation

For immunoblot assay, cells were lysed with RIPA buffer (50 mM Tris–HCl (pH 7.4), 150 mM NaCl, 1% NP-40, 0.1% SDS, 0.1% sodium deoxycholate, 5 mM EDTA, 5 mM EGTA) supplemented with protease and phosphatase inhibitor. Lysate were collected after centrifugation at 13,000 rpm for 15 min at 4 °C. The lysates were boiled for 5 min in the sample buffer. The lysates were separated by SDS–PAGE and transferred to the nitrocellulose membrane (Millipore). The blots were incubated in blocking buffer (TBS containing 5% non-fat milk, and 0.1% Tween-20) for 1 h at room temperature. And then incubated with primary antibody for overnight at 4 °C. The blots were incubated with secondary antibody for 90 min at room temperature. The immunoblots were visualized by enhanced chemiluminescence system (ECL; Amersham Biosciences). For immunoprecipitation assays, cells were lysed with E1A buffer (50 mM Tris–HCl (pH 7.4), 150 mM NaCl, 0.1% NP-40, 1 mM DTT, 5 mM EDTA). Cell extracts (500–1000 μg) were immunoprecipitated with 1 μg of indicated antibody at 4 °C for 12 h. After the antibody incubation, the cell extracts were incubated with protein A-Sepharose beads (GE healthcare Bioscience AB) for 90 min at 4 °C. Immunoprecipitated proteins were washed four times with E1A buffer. The samples were boiled with 2X SDS sample buffer for 5 min. The samples were separated by SDS–PAGE and transferred to the nitrocellulose membrane (GE Healthcare) for immunoblotting.

### Semi-denaturing detergent agarose gel electrophoresis

After transfected with determined plasmid in Huh7 cells, cells were lysed with 1X SDD sample buffer (1X TBE buffer, 10% glycerol, 2% SDS, 0.0025% bromophenol blue). The samples were separated by 2% agarose gel and running in 1X TBE and 0.1% SDS for 1 h with a constant voltage of 100 V at 4 °C. Samples were later transferred to the PVDF (Millipore) for blotting.

### Cross-linking of HBx

To detect HBx aggregates, cell extracts were incubated with 1 mM of disuccinimidyl substrate (Thermo Fisher, 21555) for 30 min at room temperature. The reaction was stopped by the addition of quenching solution at a final concentration of 10 mM Tris–HCl (pH 7.5) for 30 min at room temperature. Subsequently, 6X sample buffer that does not contain SDS and β-mercaptoethanol was added into the reaction mixture, which was separated on the SDS–PAGE and immunoblotted with indicated antibodies.

### Immunocytochemistry and confocal microscopy

Huh7 cells were seeded on coverslips in six-well plates that were transfected with plasmids and was incubated for 36 h. For visualization of mitochondria, cells were stained for 30 min with 125 nM Mito Tracker Red^TM^ Red CMXRos (Invitrogen, M7512) before harvest. The cells were then fixed with 4% paraformaldehyde in phosphate-buffered saline (PBS) for 10 min at room temperature. And then washed with a mixture of PBS and methanol. The fixed cells were permeabilized with methanol for 20 min at −20 °C. For double immunofluorescence staining, cells were blocked with 1% bovine serum albumin in PBS for 1 h at room temperature and followed by primary overnight antibody incubation at 4 °C. Cells were than washed three times and incubated with fluorescence-conjugated secondary antibody for 1 h at room temperature. The cells were washed with PBS for three times and mounted using Fluorescent Mounting Medium (Dako). The fluorescence images were captured with an LSM 510 and analyzed using LSM 510 image software (Carl Zeiss).

### Mito-Keima staining

The mt-Keima is a mitochondrially localized pH—indicating fluorescent protein previously described^28^. The method used to detect mitophagy reflects lysosomal delivery. The mt-Keima at neutral pH was detected by excitation with the 457 nm laser line, whereas the extraction at the acidic pH was detected at 561 nm. To trace mt-Keima, all experiments were performed using Huh7 cells. They were seeded in a six-well plate.

### Luciferase reporter assay

Huh7 cells were transfected with 200 ng of COX-2 luciferase reporter plasmid or NF-κB luciferase reporter plasmid and Renilla-luciferase (pRL-TK) plasmid. At the same time, the cells were treated with 200 or 400 ng of HBx-Flag or HBx-Mito-Flag. Either 400 ng Myc-MARCH5, or control (pcDNA3.1) were transfected to each of the four conditions, respectively. DNA and PEI were mixed into Opti-MEM (GIRCO-BRL) for 15 min and the incubated medium was directly added to Huh7 cells for 36 h. Luciferase activity was measured at 36 h after transfection using a luminometer (Promega) with a dual-luciferase reporter assay system according to the manufacturer’s instructions (Promega). The data represent relative firefly luciferase activity normalized to Renilla luciferase activity.

### Determination of ROS levels

Intracellular ROS levels were determined by staining with 10 mM of the ROS-sensitive dye 5-(and-6)-chloromethyl-2,7-dichlorodihydrofluorescein diacetate (DCFDA; Molecular Probes) for 20 min at 37 °C. Analysis by fluorescence-activated cell sorting (FACS) was done immediately after DCF-DA staining (FACS Vantage, Becton Dickinson). The results present the average of at least three independent experiments.

### In vivo and in vitro ubiquitination assay

Cells were treated with 10 μM of MG132 for 12 h before harvest. Whole cells were lysed with RIPA buffer (50 mM Tris–HCl (pH7.4), 150 mM NaCl, 1% NP-40, 0.1% SDS, 0.1% sodium deoxycholate, 5 mM EDTA, 5 mM EGTA) containing complete protease and phosphatase inhibitors. The same amount of protein lysates (700–1000 μg) were immunoprecipitated with anti-Flag antibody and further incubated with protein A agarose beads 4 °C for 1 h 30 min. The immune complex was washed extensively four times with RIPA buffer and boiled for 5 min with 2X sample buffer. Analysis of ubiquitination was performed by immunoblotting using anti-Ub antibody. For in vitro ubiquitination assay, immunoprecipitated Myc-MARCH5 and HBx-Flag were prepared from lysates of HEK293T cells transfected with Myc-MARCH5 or Flag-HBx, individually. Immunoprecipitates were incubated with 100 ng of E1 (Boston Biochem), 400 ng of E2 (UbcH5b, Boston Biochem), and 2 μg of ubiquitin (Boston Biochem) in reaction buffer (50 mM Tris (pH 7.4), 2 mM MgCl_2_, 4 mM ATP (Sigma), 1 mM DTT) at 30 °C for 2 h. The reaction was terminated by addition of 4X sample buffer.

### Mitochondria isolation

HEK293T cells were transfected with HBx-Flag, Myc-MARCH5^WT^, and Myc-MARCH5^H43W^ plasmid for 36 h. The cells were homogenized and cytosolic fraction was obtained after centrifugation at 13,000 × *g*. Mitochondria-containing membrane pellet was used as mitochondria fraction. Alternately, mitochondria isolation kit (Mitochondria Isolation Kit for Cultured Cells, #89874) was used.

### Statistics

Each experiment was repeated at least three times. Statistical significance was determined by comparing mean values (±standard error of the mean: S.E.M.) using Student’s *t*-test and Wilcoxon’s signed-rank test was assumed for *p* < 0.05 (*), *p* < 0.01 (**), and *p* < 0.001 (***). Overall survival analysis was estimated using the Kaplan–Meier. Statistical analysis was performed with the GraphPad Prism 5 software (GraphPad Software Inc., Sandiego, CA, USA) was used to perform the statistical analyses

## Supplementary information

Supplementary Figure Legends

Yoo_Y_et_al Supplymentary figures

Yoo_Y_et_al Supplymentary figures-1

Yoo_Y_et_al Supplymentary figures-2

Yoo_Y_et_al Supplymentary figures-3

Yoo_Y_et_al Supplymentary figures-4
