# Supplementary material for: Mitochondria ubiquitin ligase, MARCH5 resolves hepatitis B virus X protein aggregates in the liver pathogenesis
Source: Cell Death Dis. 2019 Dec 9;10(12):938. doi: 10.1038/s41419-019-2175-z (PMC6901512; doi:10.1038/s41419-019-2175-z)
Supplement: Supplementary file 1 — Supplementary Figure Legends [file 41419_2019_2175_MOESM1_ESM.docx]

**Title: Mitochondria ubiquitin ligase, MARCH5 resolves hepatitis B virus X protein**

**aggregates in the liver pathogenesis**

**Authors:** Young-Suk Yoo^1#^, Yeon-Ji Park^1, 2#^, Ho-Soo Lee^1#^, Nguyen Thi Kim Oanh^1^, Mi-Young Cho^1^, June Heo^1, 2^, Eun-Seo Lee^1, 2^, Hyeseon Cho^3^, Yong-Yea Park^4^*, Hyeseong Cho^1, 2^*

**Affiliation:** ^1^Department of Biochemistry and Molecular Biology, Ajou University School of Medicine, Suwon 16499, South Korea. ^2^ Department of Biomedical Sciences, Graduate School of Ajou University, Suwon 16499, South Korea. ^3^ Laboratory of Immunogenetics, National Institute of Allergy and Infectious Diseases, NIH, Bethesda, MD, USA. ^4^MOGAM Institute for Biomedical Research, Gyeonggi-do 16924, South Korea.

**Address correspondence to:**

Hyeseong Cho ([hscho@ajou.ac.kr](mailto:hscho@ajou.ac.kr))

Department of Biochemistry and Molecular Biology, Ajou University School of Medicine, Suwon 16499, South Korea, Phone:82-31-219-5052, FAX:82-31-219-5059

Yong-Yea Park (Yongnye@naver.com)

MOGAM Institute for Biomedical Research, Gyeonggi-do 16924, South Korea, Phone : 82-31-260-9800

^#^Y.Y. and Y.P. and H.L. contributed equally to this work.

**SUPPLEMENTARY INFORMATION**

**Supplementary Fig. 1 MARCH5 mRNA expression among patients with HBV or HCV infection.** MARCH5 mRNA expression levels in HCCs were obtained from the TCGA database. Among 138 cases, 46 cases were HCCs with HBV infection and the rest were HCCs with HCV infection. MARCH5 expression levels were not significantly different between them (NS; Not significant, *p*=0.3846 by Mann Whitney test).

**Supplementary Fig. 2 Subcellular localization of HBx and MARCH5. a** Quantification of subcellular localization of HBx-Flag in nucleus, cytosol or mitochondria **b** Myc-MARCH5 and MARCH5 mutants were transfected into HeLa cells and their subcellular localization at the mitochondria was examined in immunofluorescence staining. Myc-MARCH5 was stained with anti-Myc antibody and co-stained with Mitotracker-Red. Scale bar, 10 μm. **c** HBx-Flag and its mutants were transfected into HeLa cells and the cells were immunostained with anti-Flag antibody along with Mitotracker-Red. Scale bar, 10 μm.

**Supplementary Fig. 3 MARCH5 promotes ubiquitination of HBx protein. a** Cells were co-transfected with HA-Ub, HBx-Flag and Myc-MARCH5^WT^. The cell lysates were co-Immunoprecipitated with anti-Flag antibody, followed by immunoblotting with anti-Ub antibody. **b** In *In vitro* ubiquitination assay, Myc-MARCH5^WT^ and HBx-Flag were incubated in a reaction mixture containing ubiquitin, 100 ng of E1 and 400 ng of UbcH5b as E2. Ubiquitination levels were determined by immunoblotting with anti-Ub antibody.

**Supplementary Fig. 4 MARCH5 knockdown induces HBx accumulation and increases the colony formation. a** Chang cells were transfected with shRNA plasmids for GFP or MARCH5 along with HBx-Flag for 48 hrs and expression levels of MARCH5 and HBx were detected by immunoblotting. **b**. Chang cells transfected with shRNA plasmids for GFP or MARCH5 along with HBx-Flag were grown for 10 days. Colonies were stained with 0.1% crystal violet and visualized under a microscope.
