# Supplementary figures and images for: Mitochondria ubiquitin ligase, MARCH5 resolves hepatitis B virus X protein aggregates in the liver pathogenesis
Source: Cell Death Dis. 2019 Dec 9;10(12):938. doi: 10.1038/s41419-019-2175-z (PMC6901512; doi:10.1038/s41419-019-2175-z)

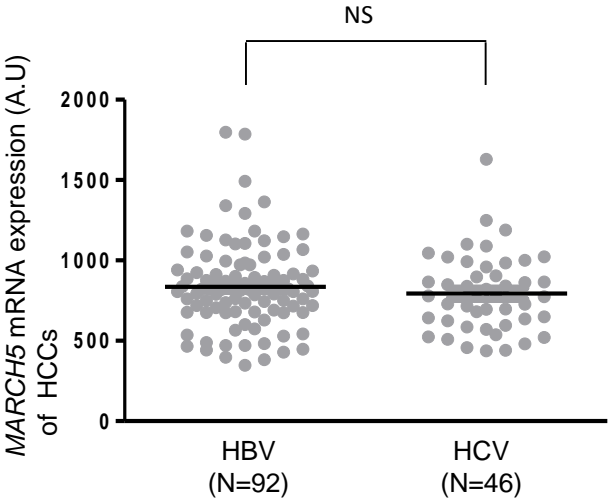

**A**

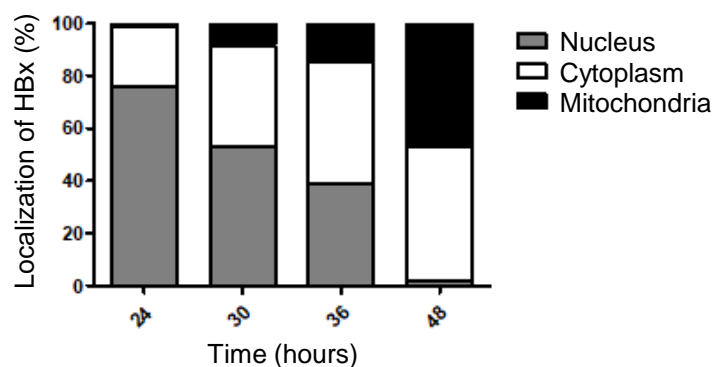

**B**

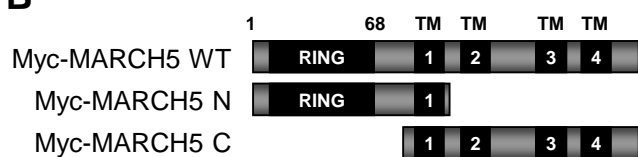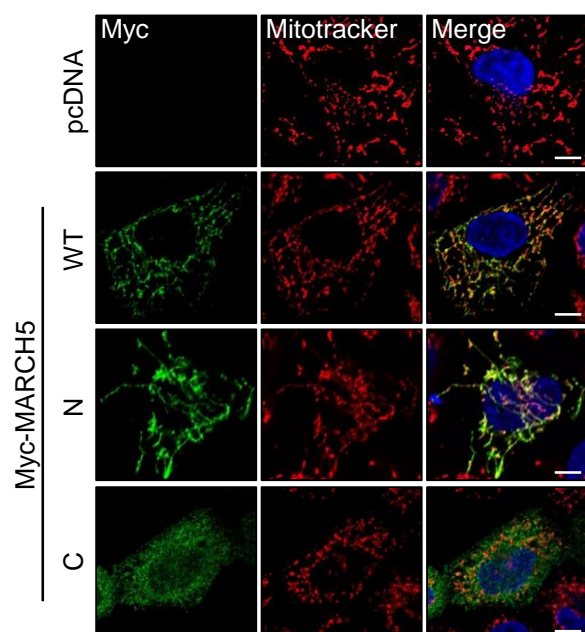

**C**

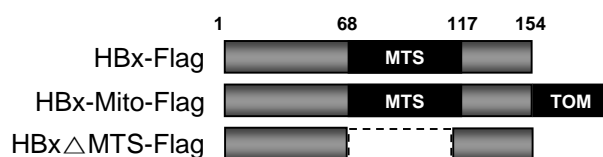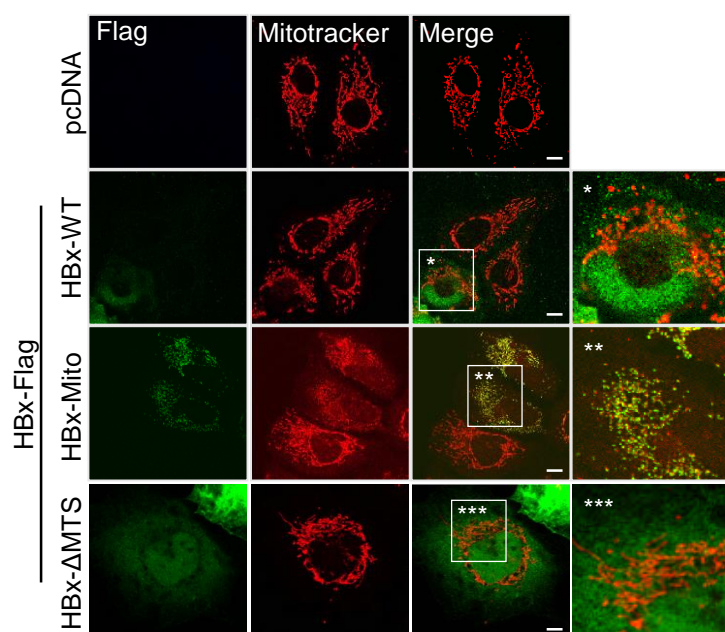

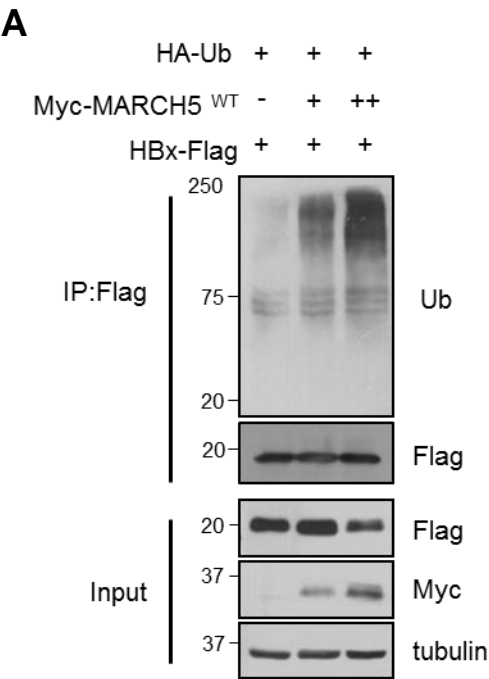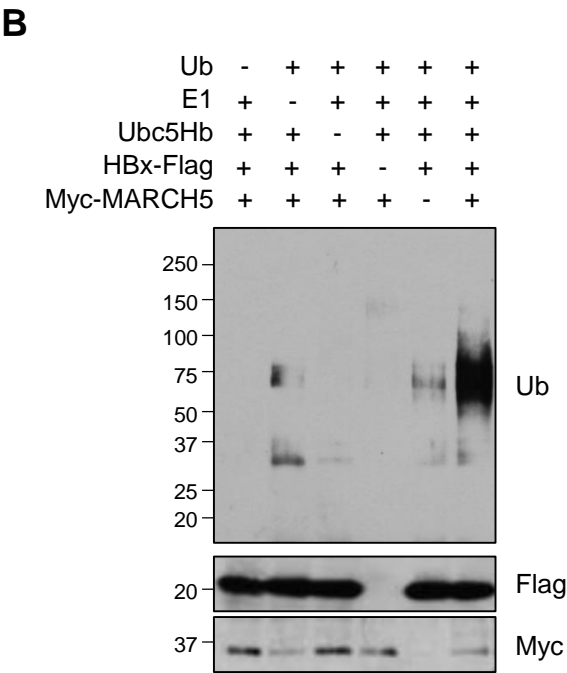

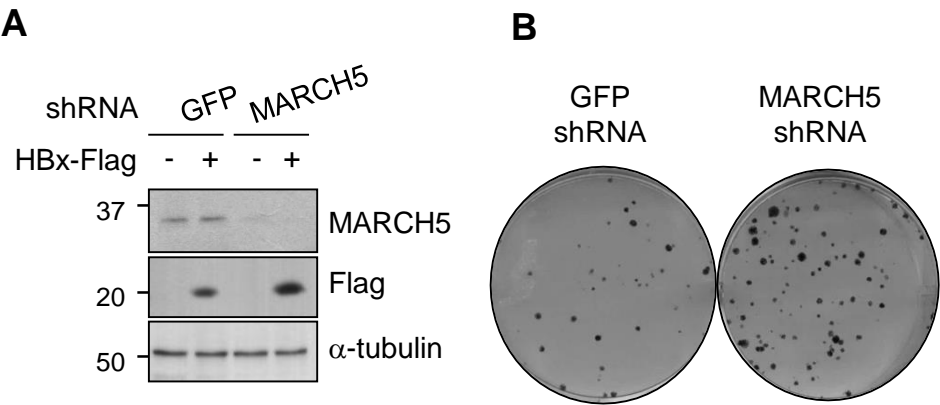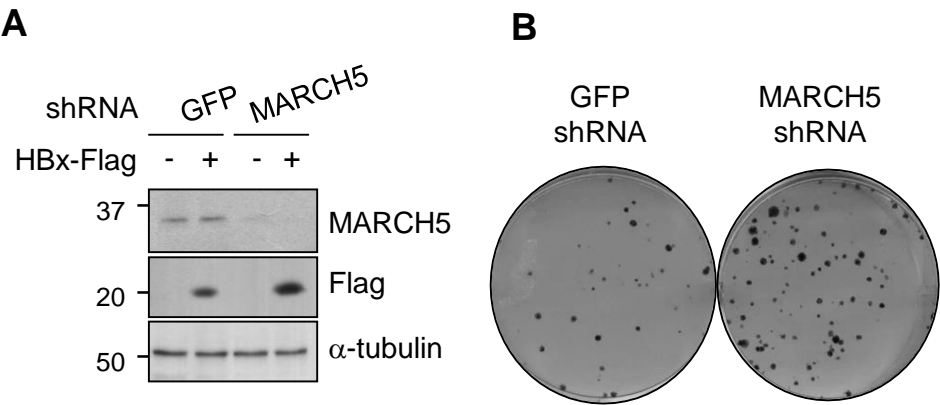

Supplement: Supplementary file 2 — Yoo_Y_et_al Supplymentary figures [file 41419_2019_2175_MOESM2_ESM.pdf]

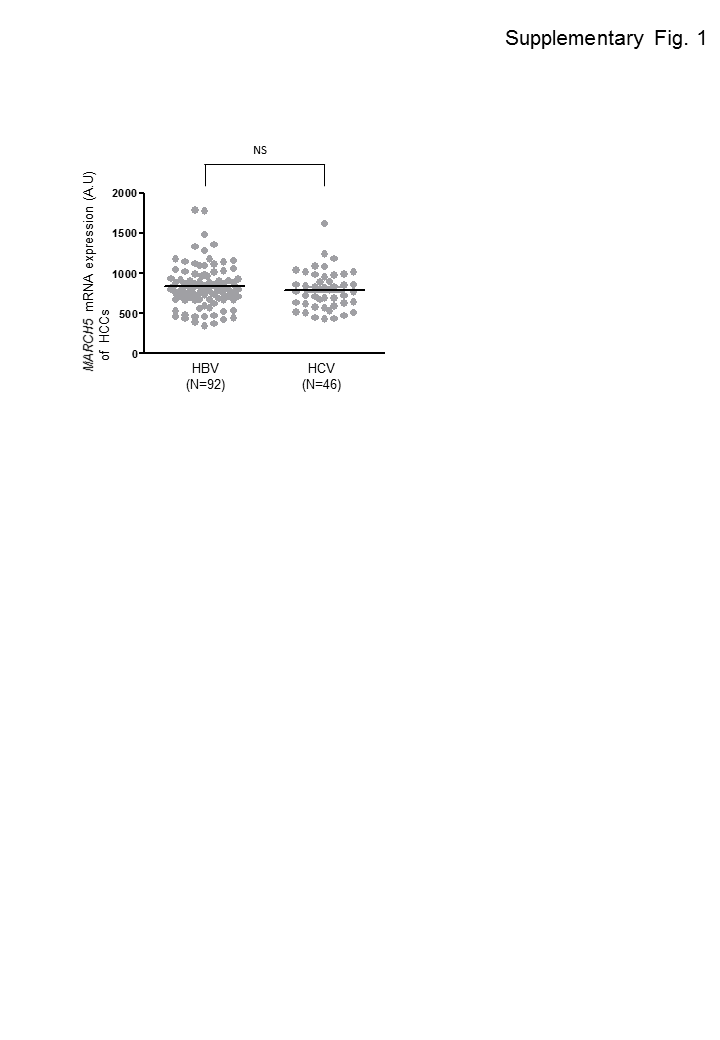

Supplement: Supplementary file 3 — Yoo_Y_et_al Supplymentary figures-1 [file 41419_2019_2175_MOESM3_ESM.tif]

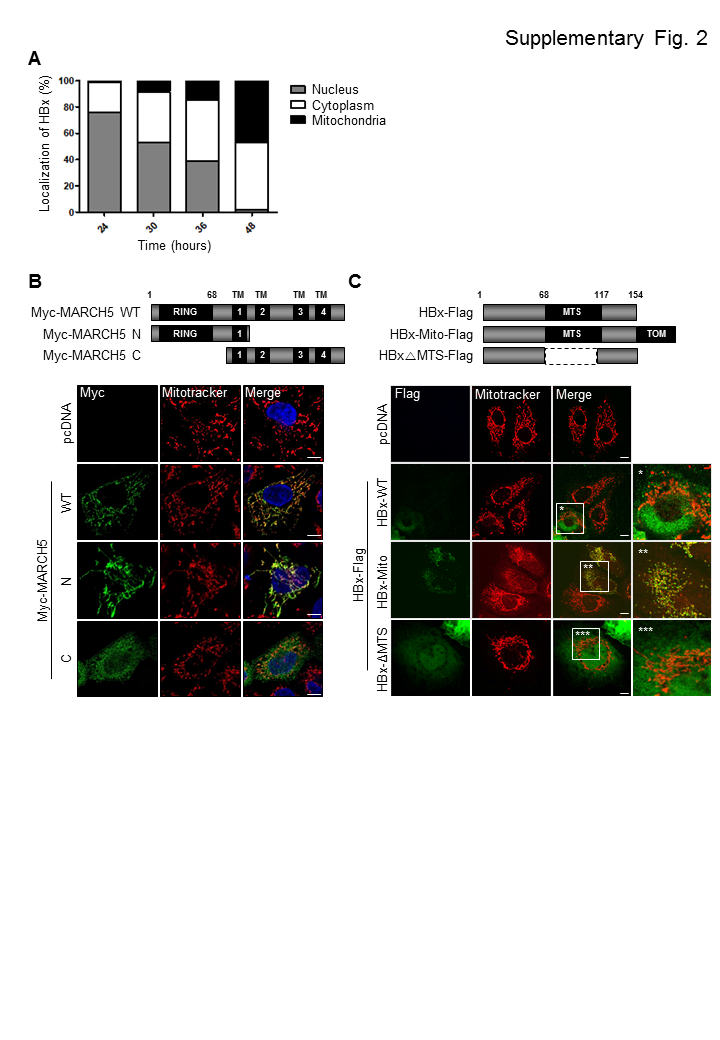

Supplement: Supplementary file 4 — Yoo_Y_et_al Supplymentary figures-2 [file 41419_2019_2175_MOESM4_ESM.tif]

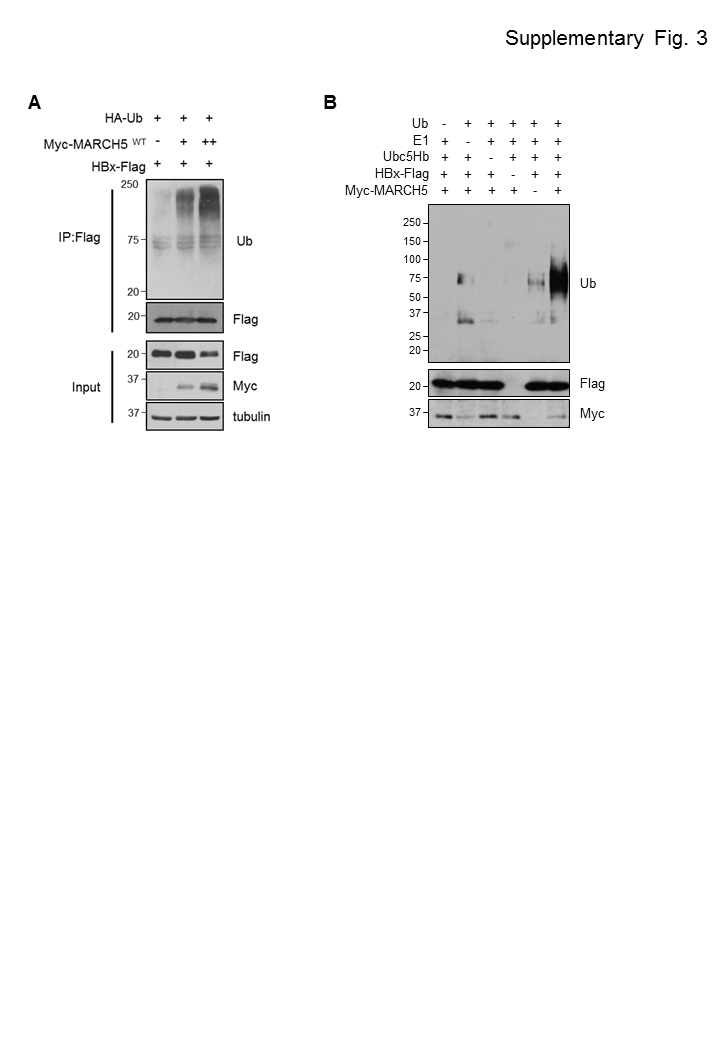

Supplement: Supplementary file 5 — Yoo_Y_et_al Supplymentary figures-3 [file 41419_2019_2175_MOESM5_ESM.tif]

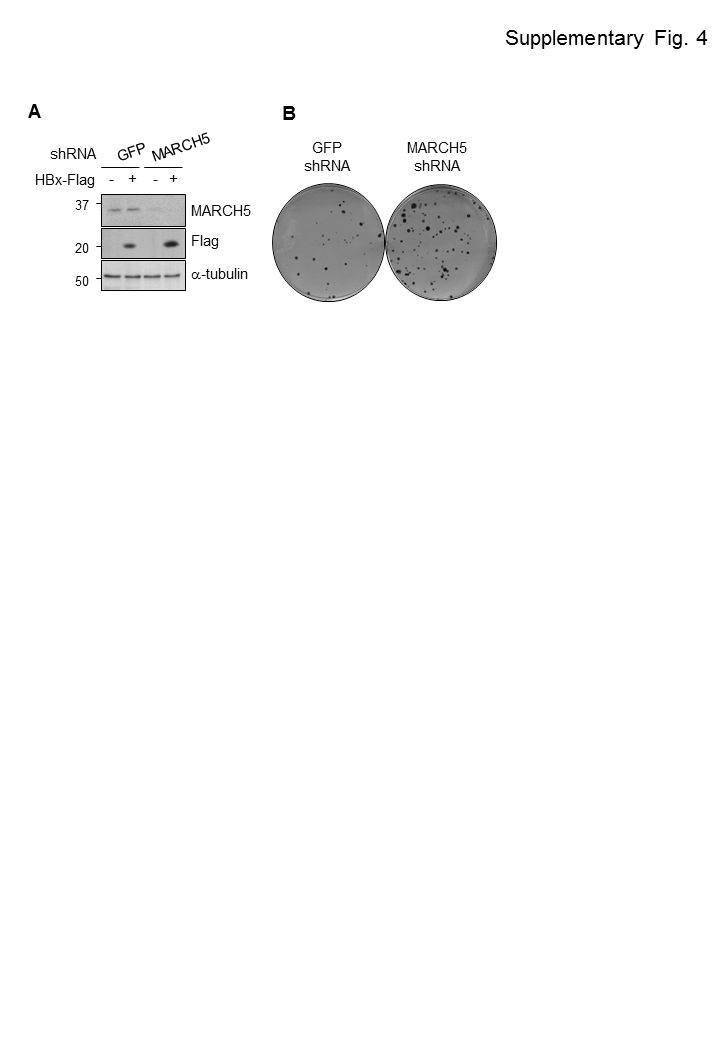

Supplement: Supplementary file 6 — Yoo_Y_et_al Supplymentary figures-4 [file 41419_2019_2175_MOESM6_ESM.tif]
